# Isolation and Characterization of an α-Glucosidase Inhibitor from *Musa* spp*.* (Baxijiao) Flowers

**DOI:** 10.3390/molecules190710563

**Published:** 2014-07-18

**Authors:** Zhanwu Sheng, Haofu Dai, Siyi Pan, Hui Wang, Yingying Hu, Weihong Ma

**Affiliations:** 1 College of Food Science and Technology, Huazhong Agricultural University, Wuhan 430070, China; E-Mails: shengzhanwu100@163.com (Z.S.); pansiyi@mail.hzau.edu.cn (S.P.); 2 Hainan Key Laboratory of Banana Genetic Improvement, Haikou Experimental Station, Chinese Academy of Tropical Agricultural Sciences, Haikou 570101, China; 3 Institute of Tropical Bioscience and Biotechnology, Chinese Academy of Tropical Agriculture Sciences, Haikou 571101, China; 4 College of Food Science and Technology, Hainan University, Haikou 570228, China; E-Mails: huiwang2013@163.com (H.W.); yingyinghu2014@163.com (Y.H.)

**Keywords:** α-glucosidase, inhibitor, hyperglycemia, flowers of *Musa* spp*.* (Baxijiao)

## Abstract

The use of α-glucosidase inhibitors is considered to be an effective strategy in the treatment of diabetes. Using a bioassay-guided fractionation technique, five *Bacillus stearothermophilus* α-glucosidase inhibitors were isolated from the flowers of *Musa* spp. (Baxijiao). Using NMR spectroscopy analysis they were identified as vanillic acid (**1**), ferulic acid (**2**), β-sitosterol (**3**), daucosterol (**4**) and 9-(4′-hydroxyphenyl)-2-methoxyphenalen-1-one (**5**). The half maximal inhibitory concentration (IC_50_) values of compounds **1**–**5** were 2004.58, 1258.35, 283.67, 247.35 and 3.86 mg/L, respectively. Compared to a known α-glucosidase inhibitor (acarbose, IC_50_ = 999.31 mg/L), compounds **3**, **4** and **5** showed a strong α-glucosidase inhibitory effect. A Lineweaver-Burk plot indicated that compound **5** is a mixed-competitive inhibitor, while compounds **3** and **4** are competitive inhibitors. The inhibition constants (*K*_i_) of compounds **3**, **4** and **5** were 20.09, 2.34 and 4.40 mg/L, respectively. Taken together, these data show that the compounds **3**, **4** and **5** are potent α-glucosidase inhibitors.

## 1. Introduction

Diabetes mellitus has become an alarming global problem in recent years. Postprandial hyperglycemia plays an important role in the development of diabetes mellitus type II and the resulting complications. One therapeutic approach to treat postprandial hyperglycemia is to retard the cleavage of glucose from disaccharide via inhibition of α-glucosidase in the digestive organs [1]. α-Glucosidase (EC 3.2.1.20) is a glucosidase that acts on 1,4-α bonds, breaking down starch and disaccharides into glucose. This enzyme is ubiquitous in plants, microorganisms, and animal tissues, although the substrate specificity of α-glucosidase differs greatly depending on the source [2]. α-Glucosidase inhibitors can decrease the postprandial increase in blood glucose and in turn help avoid the onset of late diabetic complications [3]. From this perspective, researchers have focused on finding more effective α-glucosidase inhibitors from natural materials for use as antidiabetic compounds, such as triterpene glycoside from *Acanthopanax senticosus* Harm leaves [4], flavonoid glycosides in *Microctis folium* [5] and polyphenols from green tea [6].

The banana planting area in China covers nearly 412,800 hectares, with an annual production of more than 1,085 million tons in 2012,which represents a huge economic value. Many banana flowers are produced; to date, in China they have only been used as organic material and fertilizer in the plantations. Some prior works have shown that crude extracts of banana flowers exhibited biological activity, including antihyperglycemic activity, advanced glycation end product (AGE) inhibitory activity [7], antimalarial activity [8], regulation of altered antioxidant and lysosomal enzyme activities [9] and wound-healing potential [10]. Our previous study showed that the banana flowers has tremendous nutritional value [11], antioxidant activity, and can be consumed as a food additive [12]. Despite a large body of studies on banana flowers, there is limited information on their chemical constituents. Thus, this study was conducted to determine the potential value of banana flowers as a routine and inexpensive source of useful biologically active compounds. The objectives of this project were to isolate, elucidate, and biologically evaluate phytochemicals found in banana flowers for α-glucosidase inhibitory activity. This is the ﬁrst phytochemical and biological study of flowers of *Musa* spp*.* (Baxijiao).

## 2. Results and Discussion

### 2.1. Isolation of α-Glucosidase Inhibitors

**Figure 1:**
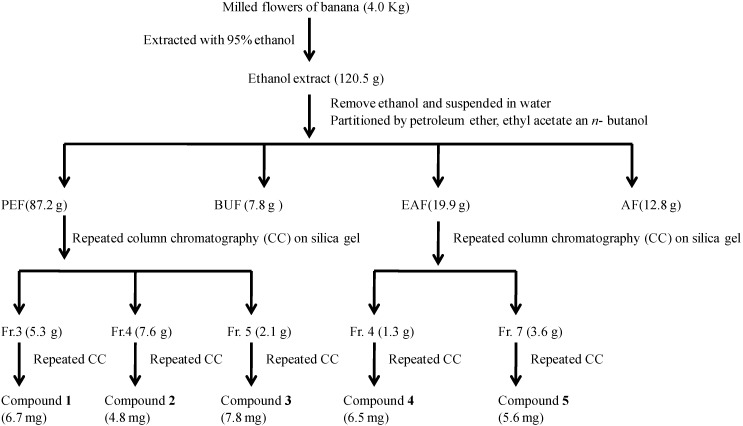
Extraction, fractionation and column chromatography separation of banana flowers.

The banana flowers were milled and extracted with 95% ethanol at room temperature. The crude extract was evaporated under vacuum, and the concentrated extracted was dispersed in water and partitioned successively with petroleum ether, ethyl acetate and *n*-butanol to obtain the PEF, EAF, BUF and AF fractions, respectively, which were all evaluated for their α-glucosidase inhibitory activities. Among the four fractions, the results suggested that the α-glucosidase inhibitory activities were mainly contained in the PEF and EAF fractions, which were therefore were subjected to successive chromatographic fractionation and purification to yield compounds **1**–**5**, as shown in Figure 1.

### 2.2. Structural Elucidation of Isolated Compounds

**Figure 2:**
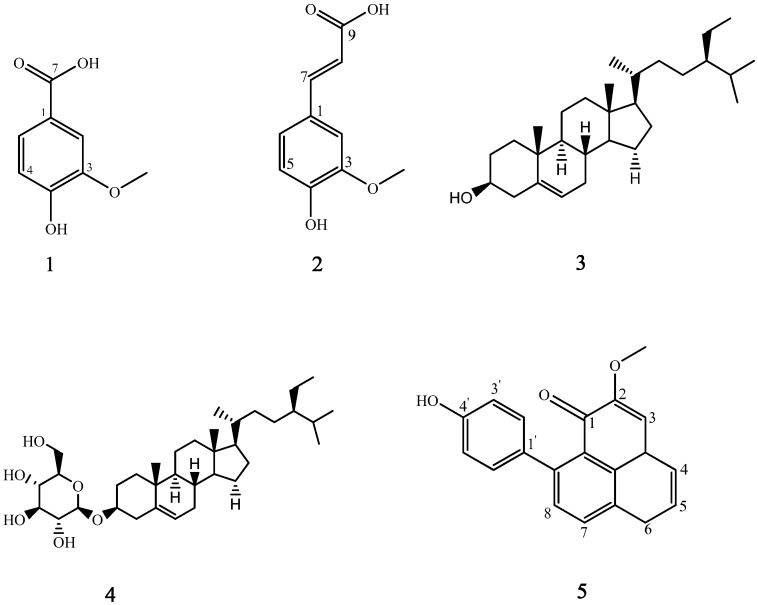
Structures of compounds **1**–**5**.

The isolated compounds were identified by one-dimensional NMR and comparison with previously reported data. The chemical structures of these isolates are shown in Figure 2. Vanillic acid (**1**) and ferulic acid (**2**) are organic acids, β-sitosterol (**3**) and daucosterol (**4**) are sterols, and 9-(4ꞌ-hydroxyphenyl)-2-methoxyphenalen-1-one (**5**) is a phenylphenalenone. This is the first report of each of these compounds being isolated from banana flowers [13].

### 2.3. α-glucosidase Inhibitory Activity of Extracts Fractionated from Flowers of Musa spp. *(*Baxijiao*)*

**Figure 3:**
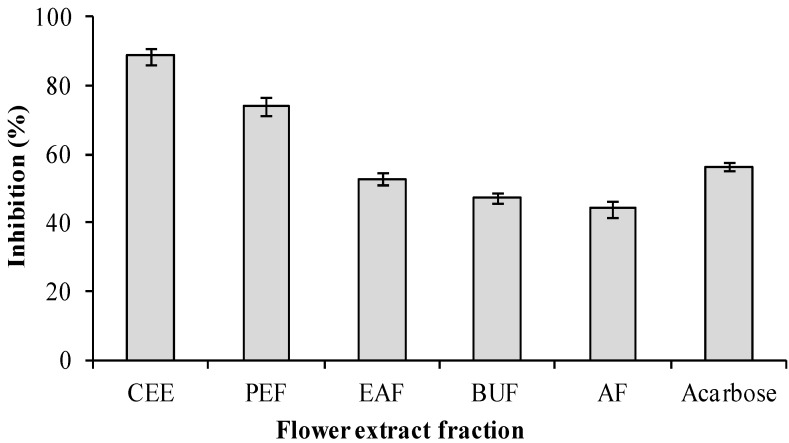
α-Glucosidase inhibitory effects of the extracts of banana flower (crude ethanol extract, CEE; Petroleum ether fraction, PEF; Ethyl acetate fraction EAF; Aqueous fraction, AF; *n*-Butanol fraction, BUF).

The inhibitory activities of the crude ethanol extract (CEE) of banana flowers were determined at the concentration of 1.5 mg/mL against α-glucosidase (Figure 3). The percent inhibition of the ethanol extract against α-glucosidase was 88.56% ± 2.3% (IC_50_ = 343.09 ± 4.35 mg/L). This result suggested that the extract of banana flower of *Musa* spp*.* (Baxijiao) might be a promising antidiabetes drug candidate. After the ethanol extract was extracted with H_2_O, petroleum ether, ethyl acetate and *n*-butanol, four fractions (AF, PEF, EAF and BUF) were obtained, and the inhibitory activities of these four extracts against α-glucosidase were also determined. Figure 3 shows that at 1.5 mg/mL, the inhibitory effect against α-glucosidase had the following order: PEF > acarbose (reference) > EAF > BUF > AF. The percent inhibition of the PEE, acarbose, EAF, BUF and AF fractions against α-glucosidase were 74.0% ± 2.5%, 56.4% ± 1.1%, 52.8% ± 1.5% and 44.1% ± 2.4%, respectively. The bioactive PEF (IC_50_ = 788.36 ± 19.32 mg/L) and EAF (IC_50_ = 1877.77 ± 12.53 mg/L) fractions were chromatographed over a silica column and further puriﬁed using Sephadex LH-20 to afford the isolated α-glucosidase inhibitors. The IC_50_ value of extract was lower than that of the fractionated compounds, because besides the isolated compounds, other phytochemical polyphenolics in the crude ethanol extracts might also contribute the α-glucosidase inhibitory activity.

### 2.4. Evaluation of α-Glucosidase Inhibitory Activity

Compounds **1**–**5** purified from *Musa* spp. (Baxijiao) showed α-glucosidase inhibitory properties, which were compared with those of acarbose, used in this study as the standard inhibitor. Acarbose decreases the hydrolysis of 4-nitrophenyl-α-D-glucopyranoside (4-NPGP) by inhibiting the action of α-glucosidase. As shown in Table 1, all of the constituents investigated exhibited a certain degree of α-glucosidase inhibitory activity, and compounds **3**, **4** and **5** showed more remarkable inhibitory effects on α-glucosidase activity than the positive control acarbose, which is commonly used for therapeutic purposes. Compound **5** in particular demonstrated excellent *in vivo* activity (IC_50_ = 3.86 mg/L). According to our results, the IC_50_ values of these compounds exhibited the following order: compound **5** > **4** > **3** > acarbose > **2** > **1**. Tabussum, *et al*. [14] reported that β-sitosterol isolated from *Chrozophora plicata* exhibited a strong inhibitory effect on α-glucosidase with an IC_50_ value of 287.12 ± 0.75 μM. Mbaze, *et al*. [15] isolated vanillic acid from the stem bark of *Fagara tessmannii* (Rutaceae), which exhibited a strong inhibitory effect on α-glucosidase with an IC_50_ value of 69.4 ± 0.8 μM. Because the inhibition is dependent on the concentration of the substrate, the enzyme and the duration of incubation with the enzyme, the α-glucosidase inhibitory effects of the same compounds in different reports are different [14]. Therefore, compounds **3**, **4** and **5** have the potential to be clinically effective α-glucosidase inhibitors.

**Table 1:** Inhibitory effects of the compounds on α-glucosidase. ^a^

Test Sample	Content (mg/L)	Maximum Inhibition (%)	IC_50_ (mg/L)
**1**	1500	18.29	2004.58
**2**	1500	42.38	1258.35
**3**	1600	98.67	283.67
**4**	1000	99.18	247.35
**5**	400	101.24	3.86
**Acarbose**	1500	56.45	999.31

^a^ All compounds were examined in a set of experiments repeated three times.

### 2.5. Mode of Inhibition of α-Glucosidase for compounds **3**, **4** and **5**

**Figure 4:**
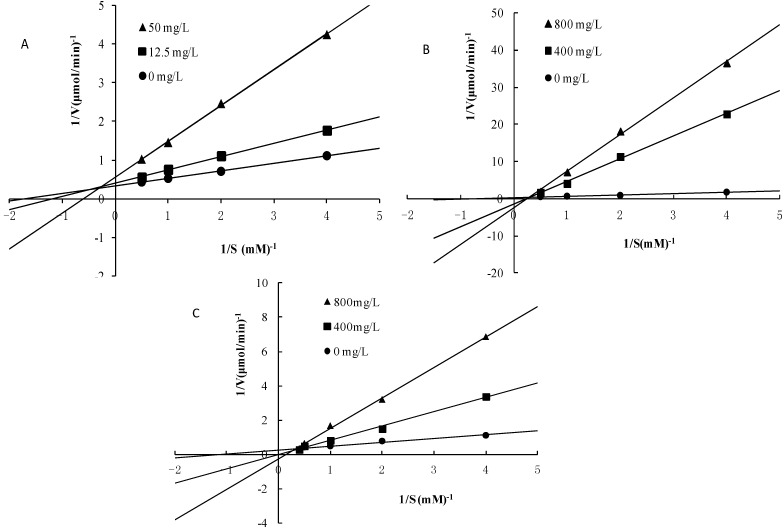
Lineweaver–Burk plot analysis of the inhibition kinetics of α-glucosidase by compounds **4** (**A**), **4** (**B**) and **4**(**C**).

The mechanism of the binding of compounds **3**, **4** and **5** to α-glucosidase was further studied. Figure 4 shows a Lineweaver–Burk plot of the α-glucosidase inhibitory activities of compounds **3**, **4** and **5** and in the absence of the inhibitor with different substrate concentrations of pNPG. It is evident from the results (Figure 4A) that the presence of compound **5** caused a decrease in *V*_max_ values compared to the control with a comparative (without much change in the *K*_m_ values) increase in *K*_m_value, indicative of typical reversible, mixed-type inhibition.

In contrast, β-sitosterol (Figure 4B) and daucosterol (Figure 4C) are competitive inhibitors. The *K_i_* value was calculated using the values of *V*_max_ obtained at 12.5 and 50.0 mg/L for compound **5** and the values of *V*_max_ obtained at 400 and 800 mg/L for compounds **3** and **4**, respectively. Compound **5** is a noncompetitive inhibitor with a *K*_i_ value of 4.40 mg/L (Figure 4). In contrast, compounds **3** and **4** are competitive inhibitors with *K*_i_ values of 20.09 and 2.34 mg/L, respectively.

## 3. Experimental

### 3.1. General Procedures and Reagents

α-Glucosidase from *Bacillus stearothermophilus* and 4-nitrophenyl-α-D-glucopyranoside were purchased from Sigma-Aldrich (St. Louis, MO, USA). The UV spectrum was recorded on a Shimadzu UV-2550 spectrophotometer (Shimadzu, Kyoto, Japan). ^1^H- and ^13^C-NMR spectra were obtained on a Bruker AV-400 instrument (Bruker, Billerica, MA, USA) using deuterated dimethyl sulfoxide (DMSO-d_6_), chloroform (CDCl_3_) or acetone (CD_3_COCD_3_) as solvents. Column chromatography was carried out on silica gel (200–300 mesh, Qingdao Marine Chemistry Company, Qingdao, China) and Sephadex LH-20 (Merck, Darmstadt, Germany) columns. All the reagents were of analytical grade.

### 3.2. Plant Materials

*Musa* spp*.* (Baxijiao), the most popular and accessible species in Hainan was chosen for this study. The banana flowers used were grown in the experimental field at the Haikou Experimental Station of the Chinese Academy of Tropical Agricultural Sciences (Chengmai City, Hainan Province, China) from January to December 2012. The plants were managed using commercial practices with standard fertilization and culture management. Mature banana plants from four plots of approximately 30 square meters per plot were collected, and the flowers were manually separated from the plant. After separation, the samples were thoroughly washed in running water, cut into small pieces, dried overnight in an air dryer at 40 °C, ground to a particle size of 40 meshes using a grinder, packed in black polyethylene bags and stored at −20 °C until further analysis.

### 3.3. Extraction and Isolation of α-Glucosidase Inhibitors

The milled flowers (4.0 kg) were exhaustively extracted with 95% ethanol (3 × 5 L, total amount 15 L) three times at room temperature for three weeks. The crude extract was obtained by filtration through absorbent gauze, and the filtrate was evaporated to dryness with a rotary evaporator under reduced pressure at 50 °C to remove the ethanol. The extract was suspended in water and extracted, respectively, with petroleum ether (PE), ethyl acetate (EA) and *n*-BuOH (BU), to offer four extracts, PE-soluble, EA-soluble, BU-soluble and residual extract fractions, and then each extract was evaporated to dryness under reduced pressure. These four fractions were designated PEF (87.2 g), EAF (19.9 g), BUF (7.8 g) and AF (12.8 g), respectively. A small amount of each fraction was redissolved in 50% dimethyl sulfoxide (DMSO) and these mixture solutions were subjected to α-glucosidase inhibitory activity assays. The PEF (87.2 g), which showed potent α-glucosidase inhibitory activity, was subjected to silica gel CC (10 × 40 cm, 0.5 kg) and eluted with a mixture of chloroform and methanol with increasing polarity (100:1, 80:1, 50:1, 30:1, 15:1, 10:1, 5:1, 2:1, 1:1, 0:1, each 1.5 L) to yield 11 fractions (Fr. 1–Fr. 11). Fractions 3 to 5, which possessed similar α-glucosidase inhibitory activity, were fractionated and studied. Repeated CC on silica gel (2.0 × 45 cm, 25 g) eluted with petroleum ether-acetone gradients (10:1–2:1, v/v,) and Sephadex LH-20 (3 × 100 cm, CHCl_3_/MeOH, 1:1, v/v) led to the isolation of compound **1** (6.7 mg) from Fr. 3 (5.3 g). Fr. 4 (7.6 g) yielded compound **2** (4.8 mg) and Fr. 5 (2.1 g) yielded compound **3** (7.8 mg). The EAF (19.9 g), which also showed potent α-glucosidase inhibitory activity, was subjected to silica gel CC (4 × 60 cm, 0.3 kg) eluted with a mixture of chloroform and methanol with increasing polarity (100:1, 80:1, 50:1, 30:1, 15:1, 10:1, 5:1, 2:1, 1:1, 0:1, each 1.5 L) to yield 11 fractions (Fr. 1–Fr. 11). Fractions 4 and 7, which possessed similar α-glucosidase inhibitory activity, were fractionated and studied. Repeated CC on silica gel (2.0 × 45 cm, 25 g) eluted with petroleum ether–acetone gradients (10:1–2:1, v/v,) and Sephadex LH-20 (3 × 100 cm, CHCl_3_-MeOH, 1:1, v/v) led to the isolation of compound **4** (6.5 mg) from Fr. 4 (1.3 g). Fr. 7 (3.6 g) yielded compound **5** (5.6 mg).

### 3.4. Spectroscopic Data

*Vanillic acid* (**1**): C_8_H_8_O_4_, colorless needles; ^1^H-NMR (acetone-*d*_6_): δ 7.59 (1H, dd, *J* = 8.2, 1.77 Hz, 6-H), 7.56 (1H, d, *J* = 1.7 Hz, 2-H), 6.91 (1H, d, *J* = 8.2 Hz, 5-H), 3.82 (3H, s, OCH_3_); ^13^C-NMR (acetone-*d*_6_): δ 167.5 (C-7), 152.0 (C-3), 148.0 (C-4), 124.0 (C-6), 122.9 (C-1), 115.5 (C-2), 113.4 (C-5), 56.3 (OCH_3_). The above data were identical to those in the literature [16]. 

*Ferulic acid* (**2**): C_10_H_10_O_4_, yellow needle;^1^H-NMR (acetone-*d*_6_): δ 6.37 (1H, d, *J* = 16.0 Hz, H-7), 7.59 (1H, d, *J* = 16.0 Hz, H-8), 6.86 (1H, d, *J* = 8.4 Hz, H-6), 7.14 (1H, dd, *J* = 8.4, 2.0 Hz, H-5), 7.32 (1H, d, *J* = 2.0 Hz, H-2), 3.92 (3H, s, OCH_3_); ^13^C-NMR (acetone-*d*_6_): δ 127.3 (C-1), 115.8 (C-2), 149.8 (C-3), 148.5 (C-4), 115.8 (C-5), 123.6 (C-6), 145.6 (C-7), 111.1 (C-8), 168.0 (C-9). The above data were identical to those in the literature [17].

*β-Sitosterol* (**3**): C_29_H_50_O, colourless needles; ^1^H-NMR (CDCl_3_): δ 0.64 (3H, s, CH_3_-18), 0.79 (3H, d, *J* = 6.4 Hz, CH_3_-27), 0.83 (3H, d, *J* = 6.4 Hz, CH_3_-26), 0.87 (3H, t, *J* = 7.2 Hz, CH_3_-29), 0.95 (3H, d, *J* = 6.4 Hz, CH_3_-21), 1.01 (3H, s, CH_3_-19), 3.51 (1H, m, H-3), 5.35 (1H, dd, *J* = 1.7, 3.5 Hz, H-6); ^13^C-NMR (CDCl_3_): δ 37.3 (C-1), 31.9 (C-2), 71.8 (C-3), 42.4 (C-4), 140.8 (C-5), 121.7 (C-6), 32.0 (C-7), 31.9 (C-8), 50.2 (C-9), 36.6 (C-10), 21.1 (C-11), 39.8 (C-12), 42.3 (C-13), 56.8 (C-14), 24.3 (C-15), 28.2 (C-16), 56.1 (C-17), 11.9 (C-18), 19.4 (C-19) , 36.2 (C-20), 18.8 (C-21), 34.0 (C-22), 26.3 (C-23), 45.9 (C-24), 29.3 (C-25), 19.8 (C-26), 19.4(C-27), 23.2 (C-28), 12.2 (C-29). The above data were identical to those in the literature [18].

*Daucosterol* (**4**): C_35_H_60_O_6_, colourless needles; ^1^H-NMR (DMSO-*d*_6_): δ 4.87 (1H, d, *J* = 7.9 Hz, H-1ꞌ), 4.55 (1H, t, *J* = 5.4 Hz, H-6ꞌa), 4.39 (1H, dd, *J* = 12.0, 5.4 Hz, H-6'b), 4.28 (1H, t, *J* = 8.0 Hz, H-4ꞌ), 4.25 (1H, t, *J* = 9.0 Hz, H-3ꞌ), 4.05 (1H, t, *J* = 8.0 Hz, H-2ꞌ), 4.00 (1H, m, H-5ꞌ), 3.95 (1H, m, H-3), 2.62 (1H, m, H-4b), 2.36 (1H, m, H-4a), 0.98 (3H, d, *J* = 6.5 Hz, CH_3_-21), 0.95 (3H, s, CH_3_-19), 0.89 (3H, d, *J* = 6.5 Hz, CH_3_-26), 0.88 (3H, t, *J* = 7.2 Hz, CH_3_-29), 0.87 (3H, d, *J* = 6.7 Hz, CH_3_-27), 0.65 (3H, s, CH_3_-18); ^13^C-NMR (DMSO-*d*_6_): δ 36.1 (t, C-1), 31.3 (t, C-2), 76.6 (d, C-3), 38.2 (t, C-4), 140.3 (s, C-5), 121.0 (d, C-6), 31.3 (t, C-7), 31.2 (d, C-8), 49.5 (d, C-9), 35.5 (s, C-10), 20.5 (t, C-11), 39.2 (t, C-12), 41.8 (s, C-13), 56.1 (d, C-14), 23.7 (t, C-15), 27.7 (t, C-16), 55.3 (d, C-17), 11.6 (q, C-18), 18.8 (q, C-19), 36.7 (d, C-20), 18.5 (q, C-21), 33.3 (t, C-22), 25.4 (t, C-23), 45.1 (t, C-24), 28.6 (d, C-25), 19.0 (q, C-26), 19.6 (q, C-27), 22.5 (t, C-28), 11.7 (q, C-29), 100.7 (d, C-1'), 73.4 (d, C-2'), 76.9 (d, C-3'), 70.0 (d, C-4ꞌ), 76.7 (d, C-5ꞌ), 61.0 (t, C-6ꞌ). The above data were identical to those in the literature [19].

*9-(4ꞌ-Hydroxyphenyl)-2-methoxyphenalen-1-one* (**5**): C_20_H_13_O_2_, red crystals; ^1^H-NMR (acetone-*d*_6_): δ 3.60 (3H, s, OCH_3_-2), 6.58 (2H, d, *J* = 8.8 Hz, H-3ꞌ, H-5ꞌ), 6.96 (1H, s, H-3), 7.30 (2H, d, *J* = 8.8 Hz, H-2ꞌ, H-6ꞌ), 7.43 (1H, d, *J* = 8.3 Hz, H-8), 7.70 (1H, dd, *J* = 8.1, 7.1 Hz, H-5), 8.09 (1H, dd, *J* = 8.1, 1.0 Hz, H-4), 8.98 (1H, dd, *J* = 7.1, 1.0 Hz, H-6), 9.33 (1H, d, *J* = 8.3 Hz, H-7); ^13^C-NMR (acetone-*d*_6_): δ 179.8 (s, C-1), 154.3 (s, C-2), 111.7 (d, C-3), 129.3 (s, C-4), 129.6 (d, C-5), 129.1 (d, C-6), 131.7 (s, C-6a), 134.7 (d, C-7), 132.4 (d, C-8), 148.2 (s, C-9), 126.4 (s, C-9a), 126.1 (s, C-9b), 134.1 (s, C-1ꞌ), 130.7 (d, C-2ꞌ, 6ꞌ), 116.1 (d, C-3ꞌ, 5ꞌ), 158.7(s, C-4ꞌ), 55.4 (q, OCH_3_-2). The above data were identical to those in the literature [13].

### 3.5. α-Glucosidase Inhibitory Activity Assay

The α-glucosidase inhibitory activities were calculated by the reported method with slight modiﬁcations [4,5,6]. One hundred microliters of 10 mg/L α-glucosidase (50 U/mg) was premixed with 0.5 mL of the test sample in 0.5 mL phosphate buffer (pH 6.8) and incubated at 37 °C for 15 min. Then 0.5 mL of 2.5 mmol/L *p*-nitrophenyl-α-D-glucopyranoside as the substrate was added to the mixture to start the reaction. The reaction was incubated at 37 °C for 15 min and stopped by adding 1 mL 0.2 mol/L sodium carbonate solution. The α-glucosidase activity was determined by measuring the release of PNP at 405 nm. For the inhibitory activity assay, crude ethanol extracts were dissolved in 50% aqueous dimethyl sulfoxide, while the chloroform, ethyl acetate, and *n*-butanol fractions and puriﬁed compounds were dissolved in dimethyl sulfoxide. Acarbose was used as positive control. The inhibition of the test sample on α-glucosidase was calculated as follows:


Inhibition (%) = {1 − (A_sample_− A_background_)/A_control_} × 100 (1)

where A_control_: Absorbance for 100% enzyme activity (+enzyme), A_sample_: Test sample (+enzyme + inhibitor) and A_background_: Inhibitor background absorbance (−enzyme + inhibitor).

### 3.6. Determination of the Inhibitory Mode of Action of the Active Compounds

To determine the kinetic mode of inhibition of isolated α-glucosidase inhibitors, Lineweaver–Burk plot analysis was performed. This kinetics study was carried out in the presence and absence of inhibitors with varying concentrations of 4-NPGP as the substrate. The mode of inhibition of the test compounds was assessed on the basis of the inhibitory effects on *K*_m_ (dissociation constant) and *V*_max_ (maximum reaction velocity) of the enzyme determined using a Lineweaver-Burk plot, which is the double reciprocal plot of the enzyme reaction velocity (V) versus the substrate (*p*-nitrophenyl β-*D*-glucopyranoside, pNPG) concentration (1/V versus 1/[pNPG]) [20].

## 4. Conclusions

The ethanol extract of banana flowers displayed α-glucosidase inhibitory activity, and the PE and EA fractions had the highest such activity. Five α-glucosidase inhibitors, including vanillic acid (**1**), ferulic acid (**2**), β-sitosterol (**3**), daucosterol (**4**) and 9-(4ꞌ-hydroxyphenyl)-2-methoxyphenalen-1-one (**5**), were isolated from PE and EA fractions. Among these compounds, β-sitosterol, daucosterol and 9-(4ꞌ-hydroxyphenyl)-2-methoxyphenalen-1-one showed excellent α-glucosidase inhibition in mixed-competitive and competitive modes, and their IC_50_ values are smaller than that of acarbose. Thus, α-sitosterol, daucosterol and 9-(4ꞌ-hydroxyphenyl)-2-methoxyphenalen-1-one have great potential to prevent hyperglycemia caused by α-glucosidase, and are thus potential candidates to treat diabetes mellitus and should be further evaluated *in vivo* studies.
